# Early efficacy of CABG care delivery in a low procedure-volume community hospital: operative and midterm results

**DOI:** 10.1186/1471-2482-5-10

**Published:** 2005-05-02

**Authors:** Thomas J Papadimos, Robert H Habib, Anoar Zacharias, Thomas A Schwann, Christopher J Riordan, Samuel J Durham, Aamir Shah

**Affiliations:** 1 Department of Anesthesiology, Medical College of Ohio, 3000 Arlington Avenue, Toledo, OH 43614, USA

## Abstract

**Background:**

The Leapfrog Group recommended that coronary artery bypass grafting (**CABG**) surgery should be done at high volume hospitals (>450 per year) without corresponding surgeon-volume criteria. The latter confounds procedure-volume effects substantially, and it is suggested that high surgeon-volume (>125 per year) rather than hospital-volume may be a more appropriate indicator of CABG quality.

**Methods:**

We assessed 3-year isolated CABG morbidity and mortality outcomes at a low-volume hospital (LVH:  504 cases) and compared them to the corresponding Society of Thoracic Surgeons (**STS**) national data over the same period (2001–2003). All CABGs were performed by 5 high-volume surgeons (161–285 per year). "Best practice" care at LVH – including effective practice guidelines, protocols, data acquisition capabilities, case review process, dedicated facilities and support personnel – were closely modeled after a high-volume hospital served by the same surgeon-team.

**Results:**

Operative mortality was similar for LVH and STS (**OM**: 2.38% vs. 2.53%), and the corresponding LVH observed-to-expected mortality (**O/E **= 0.81) indicated good quality relative to the STS risk model (O/E<1). Also, these results were consistent irrespective of risk category: O/E was 0, 0.9 and 1.03 for very-low risk (<1%), low risk (1–3%) and moderate-to-high risk category (>3%), respectively. Postoperative leg wound infections, ventilator hours, renal dysfunction (no dialysis), and atrial fibrillation were higher for LVH, but hospital stay was not. The unadjusted Kaplan-Meier survival for the LVH cohort was 96%, 94%, and 92% at one, two, and three years, respectively.

**Conclusion:**

Our results demonstrated that high quality CABG care can be achieved at LVH programs if 1) served by high volume surgeons and 2) patient care procedures similar to those of large programs are implemented. This approach may prove a useful paradigm to ensure high quality CABG care and early efficacy at low volume institutions that wish to comply with the Leapfrog standards.

## Background

For many years investigators have studied the relationship between the outcomes of high procedure-volume institutions with those of low procedure-volume institutions. This has been especially true for coronary artery bypass grafting (CABG) surgery with varied results [1-11]. Indeed, recent studies have indicated that low volume-procedure institutions performing CABG surgery may have good outcomes particularly if associated with high volume surgeons (> 125 cases per year) [12-15].

In this report we review our experience with a recently initiated small community hospital cardiac surgery program modeled after a similar high volume practice in the region. In this paradigm a group of high volume cardiac surgeons expanded their practice, protocols, and procedures to include the smaller institution. We reasoned that this paradigm can be used successfully by low volume hospitals. We tested this contention by comparing the operative and midterm results of an LVH to the national data as reported for the Society of Thoracic Surgeons (STS) National Cardiac Database over the same period.

## Methods

### Patients

We retrospectively reviewed the initial 504 patients of a new cardiac surgery program undergoing isolated CABG between February 1, 2001 – December 31, 2003 at Saint Luke's Hospital (Maumee, Ohio), a 189-bed community hospital in Northwest Ohio. The information was extracted from a local database. Patients were excluded if they had concomitant valve, other cardiac, or carotid surgery. Institutional Review Board approval was obtained for this ongoing clinical cardiac surgery database research. The requirement for informed consent was waived by the Institutional Review Board.

### Clinical data / end points

Clinical data on patients undergoing revascularization have been systematically abstracted and recorded in a dedicated cardiac surgery database, and are regularly reported to the STS national cardiac surgery database. The primary end points were operative mortality and morbidity (complications and length of hospital stay), and those were compared to the STS 2001–2003 data. In addition, LVH 0- to 3-year all-cause mortality for survival data was collected and combined via the Social Security Death Index (conducted in August 2004). Allowing for a 3-month lag, this corresponds to a follow-up between 5 and 41 months.

### Statistical analysis

Continuous data were expressed as mean ± SD. Baseline variables were compared by use of the Wilcoxon rank-sum test, t-test, or the χ^2 ^test as appropriate. Actual, risk-adjusted and observer-to-expected operative mortality (OM, Adj. OM, and O/E, respectively) data are reported as per the latest STS CABG risk model [16]. Effects of explanatory variables on OM were derived by logistic regression. Survival was compared with Kaplan-Meier analysis (log rank test) and multivariable Cox proportional-hazards regression. The latter was done to assess the effects of the varying death hazard on long-term mortality predictors and their associated risk ratios (RR). Regression model selection was done with backward elimination (Wald statistic – confirmed using forward and stepwise selection). A P < 0.05 cutoff was used to indicate significance (SPSS version 10.0, SPSS Inc., Chicago, IL).

## Results

A total of 504 CABG procedures were performed in the initial 35 months of this new cardiac surgery program. This LVH population demonstrated a similar distribution of age and gender to that of the STS, whereas race and body mass index differed significantly (Table 1). Our LVH patients had a higher incidence of three vessel disease, obesity, preoperative myocardial infarction and family history of coronary artery disease. The STS cohort exhibited more peripheral vascular disease, chronic obstructive pulmonary disease, angina, preoperative renal failure, hypercholesterolemia, congestive heart failure, and trended toward more cerebral vascular accidents (p = .06). These differences in preoperative morbidity led to a lower STS predicted mortality risk in our population, 2.94% vs. 3.13%. The LVH cohort had a relatively greater incidence of emergencies, use of internal mammary arteries, use of intra-aortic balloon pumps, and blood transfusions. The incidence of elective cases, redo surgery, and off-pump procedures were less than the STS. Total CPB (cardiopulmonary bypass) and cross-clamp times were shorter.

**Table 1:** Patient Demographics, Risk Factors and Operative Data

Variable	Study Site n mean ± SD	2001–2003 % median (25%–75%)	STS (2001–03) % median (25%–75%)	P-Value
No. of Patients	504		448841	
Demographics/Risk Factors				
Age (yrs)	64 ± 11	65 (56–72)	66 (57–74)	
Male	368	73.0	71.3	.433
Caucasian	474	94.0	87.1	<.001
Black	5	1	5.17	<.001
Hispanic/Other	25	4.96	6.84	
BSA (m2)	2.08 ± 0.25	2.08 (1.89–2.26)	1.95 (1.82 – 2.13)	<.001
Obese (BMI>35 kg/m2)	103	20.4	13.4	<.001
Current Smoker	101	20	22.1	.295
Family History of CAD	403	80	43.2	<.001
Diabetes	168	33.30	35.0	.469
Insulin-dependent	41	8.13	10.47	.102
Hypercholesterolemia	323	64	68.49	.038
Renal Failure	10	1.98	5.20	.002
Hypertension	358	71	74.78	.060
Peripheral Vascular Disease	35	6.94	15.80	<.001
Cerebrovascular Disease	60	11.90	13.21	.425
COPD	68	13.50	18.61	.004
Myocardial Infarction	266	52.80	45.53	<.001
Congestive Heart Failure	44	8.73	13.80	<.001
Unstable	222	44.10	47.05	.193
Arrhythmia (any)	42	8.30	9.43	.443
Triple Vessel Disease	422	83.70	74.63	<.001
Left Main Disease >50%	118	23.40	24.54	.594
Ejection Fraction (%)	48 ± 11	50 (40–55)	50 (40–60)	
Previous CV intervention	116	23.00	20.36	.155
				
Operative Data				
Elective	129	25.60	51.88	<.001
Emergent	63	12.50	4.10	<.001
Redo Surgery	15	2.98	8.93	<.001
No. of Grafts	3.58 ± 1.01			
Arterial	1.63 ± 0.98			
Vein	1.94 ± 0.98			
ITA Used	473	93.80	89.84	.004
Left ITA used	471	93.50	89.34	.004
Aortic Cross-Clamp (min)	58 ± 22	55 (45–68)	63 (47 – 83)	.004
Perfusion time (min)	90 ± 31	85 (70–106)	94 (73 – 119)	
Off-pump	21	4.17	20.48	<.001
				
STS Predicted Mortality (%)	2.94 ± 5.0		3.13	

Ejection fraction was available in 464 patients. CV intervention = any cardiovascular intervention (surgery, angioplasty, or stenting). The urgent cases (197/504) are not compared in the STS database.

Our LVH operative mortality (OM) did not statistically differ from the STS (2.38% vs. 2.53%). The corresponding O/E morality ratio was 0.81 and adjusted OM was 1.9%. Multivariate OM predictors were age, emergency status, and time on cardiopulmonary bypass (Table 2). The patients in the very low risk (<1%) and low risk (1–3%) categories fared better than the STS with actual mortality rates and O/E mortality ratios of 0%, 0.0 and 0.9%, 0.52, respectively. The moderate to high-risk category (>3%) had an actual mortality rate (7.3%) and an O/E mortality ratio (1.03) that were comparable to the STS.

**Table 2:** Multivariate predictors of operative mortality by logistic regression applied to 504 patients

						95%C.I.	
Variables	B	S.E.	Wald	Sig.	OR	Lower	Upper
Age (yr)	.092	.035	6.942	.008	1.096	1.024	1.173
Emergency	1.312	.674	3.788	.052	3.712	.991	13.908
Time on CPB (min)	.026	.007	13.421	.000	1.027	1.012	1.041
Constant	-12.977	2.788	21.673	.000	.000		

Compared to the STS data, post-operative complications of leg wound infection, prolonged ventilation, renal dysfunction (no dialysis), and atrial fibrillation occurred more frequently at our LVH (Table 3). Also, LVH had a greater rate of 30-day readmissions. However, total length of stay, post-operative length of stay, post-operative ventilator hours, strokes, sepsis, pneumonia, urinary tract infection, and sternal wound infection were similar.

**Table 3:** Operative Outcomes

Variable	Study Site n mean (SD)	2001–03 % median (25%–75%)	STS (2001–03) % median (25%–75%)	P – value
Intra-aortic Balloon pump (any)	73	14.5	9.2	<.001
Blood Transfusion (Any)	256	50.8	45.3	.014
				
Complication				
ReOp Bleeding	14	2.78	2.50	.798
Perioperative myocardial infarction	3	0.60	1.07	.414
Sternal wound infection	1	0.20	0.47	.578
Leg wound infection	14	2.78	0.70	<.001
Septicemia	7	1.39	1.03	.570
Urinary Infection	9	1.79	1.67	.973
Permanent Stroke	4	0.79	1.53	.242
Transient Stroke	7	1.39	0.93	.406
Post-Op Ventilator (hours)	23.2 80.3	6.3 (4.0–15.8)	21.73	
Ventilator Prolonged (>24 hrs)	53	10.5	7.5	.012
Pneumonia	15	2.98	2.87	.989
Renal Failure	34	6.75	3.47	<.001
Atrial Fibrillation	144	28.6	19.9	<.001
				
Operative Mortality	12	2.38	2.53	.940
Total LOS (days)	8.59 6.11	7 (5–10)	8.97	
Post-Op LOS (days)	6.45 5.27	5 (4–7)	6.87	
30-day Readmission	59	11.7	8.4	.009

**Figure 1:**
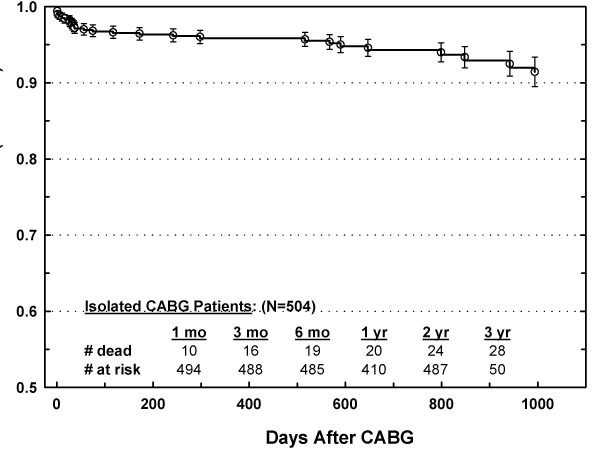
Kaplin-Meyer survival curve for 504 LVH CABG patients. Bars = standard error.

**Figure 2:**
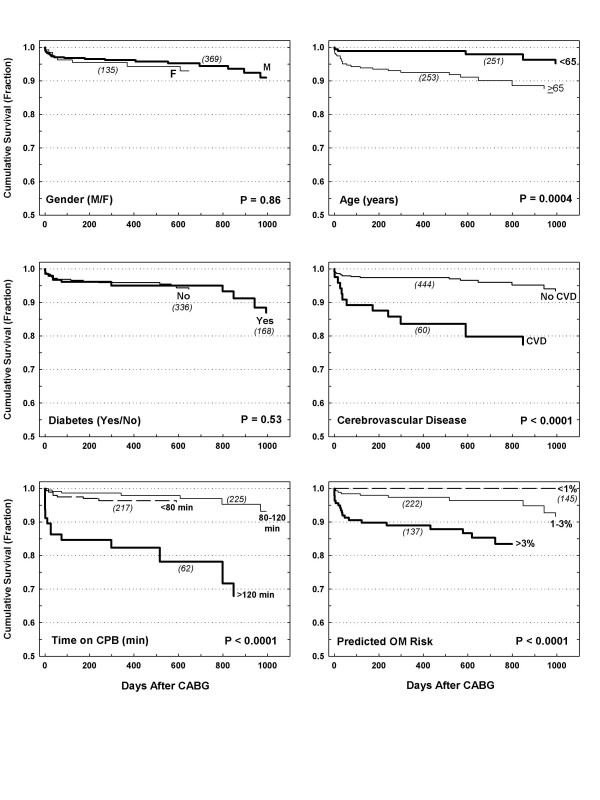
Effects of gender, age, diabetes, cerebrovascular disease, time on CPB, and operative mortality (OM) on midterm survival. P value reflects log-rank test results.

Unadjusted Kaplan-Meier survival was 96%, 94 %, and 92% at 1, 2, and 3 years, respectively (Figure 1), which was comparable to the STS. Effects of six patient variables on survival are shown in Figure 2. Gender and diabetes did not affect midterm survival, while survival for older (> 65 years) patients, cerebral vascular disease, longer time on CPB, and higher STS predicted operative mortality risk all exhibited significantly worse midterm survival.

Predictors of 0–3 year mortality were derived by multivariate (proportional hazard) Cox regression analysis and included longer time on CPB (RR = 1.15), all vein grafts (RR = 5.74), cerebral vascular disease (RR = 3.79), age (RR = 1.63 per 10 years), redo surgery (RR = 3.46), and congestive heart failure (RR = 2.48). Preoperative renal failure approached significance (RR = 3.81, p = .078) (Table 4).

**Table 4:** Predictors of 0 – 3 year mortality derived by Multivariate (proportional hazard) Cox regression analysis.

Variables	B	SE	Wald	Risk Ratio	P-value	95%	C.I.
Time on CPB (per 10 minutes)	0.02	0.00	16.26	0.0001	1.15	1.08	1.23
All vein grafts	1.75	0.55	10.15	0.0014	5.74	1.96	16.82
Cerebrovascular Disease	1.33	0.43	9.82	0.0017	3.79	1.65	8.72
Age (per 10 years)	0.06	0.02	8.28	0.0040	1.63	1.20	1.91
Redo Surgery	1.24	0.55	5.00	0.0254	3.46	1.17	10.26
Congestive Heart Failure	0.91	0.45	4.07	0.0435	2.48	1.03	6.00
Pre-operative Renal Failure	1.34	0.76	3.11	0.0779	3.81	0.86	16.86

95% C.I = 95% confidence interval; CPB = cardiopulmonary bypass;

Predictors are arranged by decreasing Wald statistic

## Discussion

While many researchers have found hospitals with higher CABG volumes to be associated with better outcomes, there has been significant interest in the potentially confounding influence of cardiothoracic surgeon procedure-volumes on this association [1,2,5,11,13-17]. Four states (California, New York, New Jersey, and Pennsylvania), which represent a quarter of the US population, have rigorous reporting systems for CABG procedures based on risk-adjusted mortality alone. In these states the Leapfrog standard includes only those hospitals in the top quartile. These "top" hospitals had overall mortality rates of 1.7% compared with 4.1% in the lower quartiles. In the rest of the nation, volume (>450 cases per annum) and mortality rates below 2.7% comprise the Leapfrog standard [18]. The use of standard risk-adjusted mortality rates may become the benchmark if rigorous outcomes measurement systems are implemented throughout the United States. Risk adjustment makes surgeon procedure volume very important. In this way, certain cases may be restricted to particular procedure volume surgeons, thus patient redistribution to regional centers may not be necessary.

Three recent studies of note have highlighted the critical importance of surgeon procedure volume [13-15]. Peterson et al found that hospital procedure-volume, as a quality marker in CABG surgery, was to be only modestly associated with risk-adjusted CABG mortality rates [14]. In fact, these researchers clearly identified many low-volume hospitals with low mortality rates and several high volume centers with higher than expected rates. In addition, Wu et al indicated that high volume cardiothoracic surgeons were associated with a lower risk of death for both low-risk and moderate-to-high risk CABG patients [15] and Birkmeyer et al demonstrated that the observed associations between hospital volume and operative mortality were modulated by surgeon volume [13].

The establishment of the STS National Cardiac Database has facilitated quality of care comparisons of cardiac surgery programs relative to national statistics [19-21]. The Center for Medicare and Medicaid Services (CMS) and the Leapfrog group have suggested that hospital volume be used as the indicator of the quality of CABG outcomes [22-24]. However, an insightful review of the evidence by Shahian and Normand lends merit to the argument that CABG surgery is so pervasive that its well-recognized techniques and well-understood pathophysiology make provision for its transportability to low volume institutions in the hands of skilled, high volume surgeons [25]. The recent Peterson et al investigation of the STS database accounted for clinical factors, differences in site variability and clustering within sites. They documented that (1) low volume institutions tended to operate more often on patients under emergent conditions, (2) the association between hospital volume and mortality was different for younger (<65 years) vs. older (>65 years) patients, (3) the hospital volume and outcome associations were confounded by the concomitant effect of surgeon volume, and (4) hospital volume per se was a poor predictor of CABG outcome [14].

In view of the above studies the issue of volume as a "proxy" yields itself to discussion. Some have criticized hospital volume as a crude indicator of surgical quality in that hospital volume is only a proxy for low mortality (high quality) [26-29]. Volume must be examined from the perspective of, not only the hospital, but also of the surgeon. The procedure volume of surgeons is an important proxy for quality CABG care. In this light, our example of a high quality, low volume center should not be unexpected, especially when our LVH used high volume surgeons.

Our study site was a small, low procedure volume hospital located in an increasingly affluent suburban community with a large rural catchment area. This accounted for the large number of Caucasians and the under-representation of African-Americans. The increased body surface area and excessive obesity that occurred in our population was to be expected according to findings that indicate Ohio is near the leading edge of the obesity experience in the United States [30,31]. The study site patients had more significant family histories of coronary artery disease (CAD), but we cannot comment as to the extent of the effect of genetic predisposition versus social impact (over-eating, smoking, lack of exercise) had upon this finding. However, the extent of obesity, and family histories of CAD could account for the significant deviation from the STS in regard to triple vessel disease and history of myocardial infarction.

The relatively recent initiation of this cardiac surgery program and its rural catchment area may account for the distribution of cases (fewer elective and redo surgeries, but more emergent cases) because: (1) the cardiac surgery program was actually put into place to support interventional cardiology, (2) the cadre of cardiologists was not as well established at this location and had fewer elective cases posted, (3) the proximity to several rural hospitals allowed rural emergent cases (whether initially presenting to the study site emergently or becoming emergent during their rural hospital stay) to receive expeditious care, (4) the surgeons did not have an office in the immediate area and this affected their contribution of elective cases to the institution, and (5) a newly established program would have less opportunity to perform redo surgeries.

The surgeons at the study location were very experienced and were strong advocates of mammary and radial artery use [32], and this is reflected in their shorter aortic cross-clamp (perfusion) times and their use of internal mammary grafts as compared to the STS. However, the surgical group was less enthusiastic for the use of off-pump coronary artery bypass procedures. The increased use of intra-aortic balloon pumps and blood transfusions in our population may be attributed to the increased severity of illness as represented by an increased percentage of emergent cases, preoperative myocardial infarctions, and triple vessel disease.

The postoperative complications of renal dysfunction (no dialysis), prolonged ventilation greater than 24 hours, leg wound infection, atrial fibrillation, and 30-day readmission deviated from the STS. Postoperative renal dysfunction was not associated with an increased need for dialysis. The higher rate of renal dysfunction may be linked to a higher incidence of emergent cases done soon after cardiac catheterization. Such prompt surgeon response may not allow sufficient time for elimination of the contrast dye that may exacerbate the associated nephropathy [33].

Postoperative atrial fibrillation in our population is greater than the STS, but is within the range reported by others [34,35]. This may be related to the greater incidence of emergent cases and shorter catheterization-to-surgery times. The latter may have compromised the efficacy our implementation of the amiodarone prophylaxis protocol in high-risk atrial fibrillation patients.

The prolonged rate of postoperative ventilation may be a function of anesthesiology practice. In the evening the cardiothoracic anesthesiologist covers the intensive care unit by beeper. Although protocols are in place regarding extubation and the anesthesiologist can be consulted at any hour, we have found that there is reluctance to extubate patients between midnight and 0600 on the part of the staff. However, this did not influence the length of stay or the rate of pneumonia.

The cardiovascular surgical assistants hired at the study location initially had limited experience with the surgical care of leg wounds. Their ability to care intra-operatively and post-operatively for leg wounds has improved and is under a continuous quality improvement process. The extent of obesity and emergent cases in our population may be a contributing factors to poor wound healing. Also, the emergent cases arrive in the operating room on many occasions after administration of antiplatelet drugs and this may contribute to postoperative hematoma formation and infection at the leg wound sites.

An increased 30-day readmission rate of the study site was also noted. The surgeons initiating this cardiac surgery program had heightened awareness as to potential patient complications in a "new" program and did not hesitate to readmit a patient of questionable physiologic status.

In our paradigm a group of high volume surgeons from a high volume hospital (HVH) established a new LVH cardiothoracic surgery program providing practice guidelines, protocols and data acquisition capabilities. In addition, the physical layout of the cardiac surgery wing and the organization of the cardiovascular intensive care unit (CVU) within a dedicated heart center were of paramount importance. It included two cardiac catheterization suites with a 10 bed holding area, two operating suites were immediately adjacent to an eight-bed CVU used as intensive care, step-down and floor beds if capacity permitted ("one-stop" for all patients). An experienced critical care nursing staff was recruited from the HVH. In addition, two anesthesiologists/intensivists with transesophageal echocardiography skills were dedicated exclusively to the heart center. They were responsible for the pre-, intra-, and postoperative critical care of all the patients. The CVU model was one that involved the cardiac surgeon as the primary physician; however the anesthesiologists were consulted on every case to support the global care given to the patient. This was particularly beneficial to the surgeons because their practice involved three institutions in addition to their office practice. Also, daily group rounds included a surgeon, anesthesiologist, nurse, respiratory therapist, and pharmacist. Each month a meeting was held, not only for case review, but to assess the program's performance against the STS database.

Clearly, surgeon procedure-volume examination will cause controversy [36-38], but studies of risk-adjusted data indicate that surgeon volume is of significant importance [13,14,16,17]. In the setting of a LVH where there are more emergent and high risk patients [14] there should be a continuous quality improvement effort in place to ensure "best practice", evidence-based care is offered to patients [12].

## Conclusion

We demonstrated that high quality CABG care with good outcomes can be achieved at a LVH program provided that that it is served by high volume cardiac surgeons and backed up by a highly trained, dedicated support team, and a sophisticated data acquisition capability and review process. This approach may prove a useful paradigm to ensure high quality CABG care and early efficacy at low volume institutions that wish to be compliant with Leapfrog recommendations.

## Competing interests

The author(s) declare that they have no competing interests.

## Authors' contributions

Drs. Papadimos and Habib wrote the abstract, methods, background, discussion, results, discussion and conclusions. Drs. Zacharias, Schwann, Riordan, Durham, and Shah assisted in writing the results section.

## Pre-publication history

The pre-publication history for this paper can be accessed here:


